# Assessing the shade matching accuracy among dental students through visual and instrumental methods

**DOI:** 10.12669/pjms.40.8.9491

**Published:** 2024-09

**Authors:** Muhammad Adeel Ahmed

**Affiliations:** 1 Muhammad Adeel Ahmed Department of Restorative Dental Sciences, College of Dentistry, King Faisal University, Al Ahsa, P.O. Box 31982, Saudi Arabia

**Keywords:** Spectrophotometer, Visual method, Correcting light, Male dental student

## Abstract

**Objectives:**

To evaluate the shade selection accuracy of male dental students according to the three dimensions of color: value, hue, and chroma, using visual and instrumental shade selection methods under different lighting conditions.

**Methods:**

This comparative cross-sectional study was instigated amongst 70 male 4^th^, 5^th^, and 6^th^ years BDS students of the Dental Clinics Complex at King Faisal University, Saudi Arabia for a duration of two months. First, the principal investigator determined the shade of tooth 11 and 46 on patient utilizing the spectrophotometer after calibration. In the next step, students were asked to select the shade of same tooth 11 and 46 using VITA 3D-Master shade guide under clinic lightening condition and corrected light. The characteristics of the color such as hue, chroma and value were recorded. The Kruskal-Wallis test was applied to find the significant difference in shade selection between visual and instrumental methods with respect to academic years.

**Results:**

The mean of the value of instrumental shade selection of tooth # 46 was slightly higher among 4^th^ year students (4.41±0.73), than that of 5^th^ year (3.72±1.02), and 6^th^ year students (3.83±0.83), with a statistically significant difference among them (p= 0.024), indicating 6^th^ year students selected darker shades. Additionally, a statistically significant relationship was noticed among 4^th^, 5^th^, and 6^th^ year students with respect to the means of the chroma of 2.18 ±1.29, 2.92±1.11, and 3.13±1.10, respectively (*p*= 0.025).

**Conclusion:**

The selection of posterior teeth shades was notably influenced by academic years, employing both instrumental and visual methods, while considering color parameters.

## INTRODUCTION

In the past few decades, there has been a significant increase in the importance of aesthetics among patients and dental professionals. It is imperative to provide an aesthetically pleasing restoration that harmoniously integrates with the patient’s surrounding teeth.^1^ Nevertheless, it is difficult to get a close shade match between the restoration and natural teeth due to the wide variety of natural tooth shade. Every dentist should be aware of the shade selection process in order to achieve the best results, as many restorations are unsuccessful because of incorrect shade selection. The human brain can identify approximately one million shades, and recently, highly precise tools capable of differentiating between ten million and one million shades have been developed. The shades of human teeth vary greatly; electronic instruments can distinguish between over 100,000 dental shades, whereas the human eye can distinguish between about 1% of these shades.^2^

Artificial tooth shade matching is influenced by the light source, the thing that has to be observed, and the observer.^3^ In a dental practice, there are three types of light available: highly variable natural daylight; the operating light of a dental unit, which leans more towards the red end of the visible spectrum than does natural sunlight; and lastly, fluorescent ceiling lights, which, in contrast to incandescent lights, have different color-rendering qualities based on the designated color temperature.^4^ A light with a color temperature of between 5,500 K and 6,500 K and a Color Rendering Index (CRI) of at least 90 offers the best conditions for matching tooth shades. It is best to match the shade of the teeth in the presence of a single light source when making this assessment because several light sources that overlap can encourage metamerism.^4^ Color-corrected lighting tubes and handheld light-correcting devices have been recommended as a way to reduce the impact of ambient lighting on matching dental shades.^5^

A study conducted in the UK examined the shade recording abilities of dental students using traditional methods, both with and without a color-correcting device, compared to a digital shade recording device. The findings indicated that the use of a color-correcting device enhanced the students’ ability to match shades more accurately than traditional methods under normal lighting conditions.^6^ Similarly, Corcodel *et al*. investigated the color matching under daylight lamp and natural daylight. They found that when compared to natural daylight, a typical daylight bulb significantly improves the ability to match shades.^7^

Another study clearly supports the idea that shade-matching abilities in corrected light sources were significantly better than in natural or clinical light.^8^ For those with poor color vision, even a low-temperature light source significantly improves color matching.^9^ Likewise, a different study examined visual shade selection under natural daylight, a dental operating light and a color-corrected light. They revealed that the best conditions for improving shade matching are achieved using a light-correcting device.^10^ For the purpose of producing closely adjusted spectral reflectance curves (optical properties) of natural and restored materials, dental professionals and technicians should ideally work in a comparable balanced, full-spectrum lighting environment. This will ensure an excellent color match and negligible metamerism.^1^

The most frequent method for visually matching tooth colors is to use dental shade guides, recognized as color standards.^3^ These are tabs with various hues that help establish the exact shade of a tooth. Numerous shade guides have been recognized including the Vitapan 3D-Master shade guide, the Hayashi shade guide, the Clark shade guide, the Chromascope shade guide, the VITA classic shade guide, and Spectatone. The most widely used and well-accepted shade guide is the VITA classical shade guide. It contains sixteen tabs in total, divided into four categories based on color.^11^ Tooth color can be measured objectively by placing a device that eliminates the negative visual illusion and functions as an observer, or subjectively by the operator using shade guides.^12^ This method yields precise and repeatable results. A spectrophotometer is one of these tools.^2^ A spectrophotometer is an advanced instrument with multiple setups that gauges an object’s spectrum reflectance. This type of photometer measures light intensity by determining the wavelength based on color. The white light source in a spectrophotometer can be an LED lamp or a tungsten filament bulb, and it produces light with a wavelength of 400–700 nm. When light enters an object through a prism, it emerges as a spectrum of wavelength bands between 10 and 20 nm. For every visible wavelength band, the amount of light that an object emits or transmits across it is measured.

The detector amplifies and displays the electrical signal it creates from the intensity of light at a certain wavelength on the device’s screen. Typically, the shade guide converts these measurements to a comparable shade tab.^2^ Nonetheless, more recent models of spectrophotometers with monochromators and photodiodes are able to measure an object’s color’s reflectance curve every 10 nm or less. Spectrophotometers can be divided into two categories based on the measurement geometry: full tooth surface measurement and spot measurement, with some variations in illumination sensors, filters, and angle of irradiance or reflection.^3^

Dental practitioners are concerned about the inherent subjectivity of visual shade matching.^5^ Various measuring tools have been developed to aid in selecting the correct tooth shade and achieving more aesthetically pleasing results. However, there is not much consensus on which tooth shade selection method provides the most consistent outcomes. We believe dental students are an ideal group to test shade-matching abilities because they are generally young adults within the same age range, have minimal experience in shade selection, and are less likely to have systemic conditions that affect color perception. Therefore, the aim of this study was to evaluate the shade selection capability of male dental students according to the three dimensions of color: value, hue, and chroma, using visual and instrumental shade selection under different lighting conditions.

## METHODS

This comparative cross-sectional study was conducted among male undergraduate BDS students at various academic levels at the Dental complex of King Faisal University, Saudi Arabia for a duration of two months. After obtaining written informed consent, 70 male undergraduate students from various academic levels participated in this study. All students were verified for color deficiency using Ishahara’s test.

### Inclusion & Exclusion Criteria:

**Fig.1:**
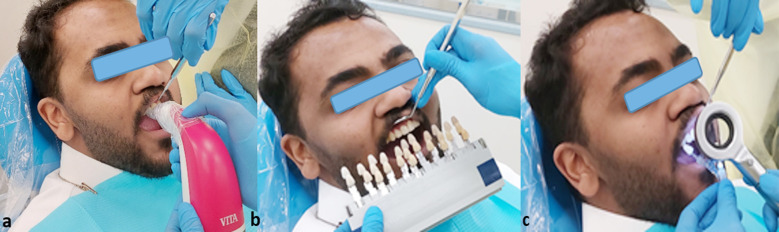
**(a)** Shade selection using Vita Easyshade® V **(b)** Shade selection under clinical lightening condition. **(c)** Shade selection under handheld corrected light (Tri-Shade, Zhengzhou, China).

**Fig.2:**
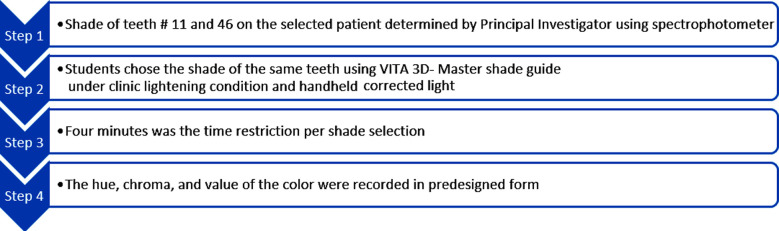
Summary of a study methodology.

The patient samples were selected using the convenience sampling technique. The principal investigator selected the patient who came for the restorative procedure and who also gave his or her permission to be included in the shade matching study. For the shade matching, teeth # 11 and 46 were chosen. On the other hand, patients with extremely poor oral hygiene, the existence of any genetic enamel or dentinal defects, patients who had previously undergone whitening procedures, patients who were using any type of orthodontic appliances at the time of the shade determination, cases lacking 11 or 46 numbered teeth, cases with prior restoration or inherent discoloration, non-vital teeth, and students with color vision deficiencies were excluded. The principal investigator first used the spectrophotometer (Vita Easyshade®V Compact Vita, Zahnfabrik, Bad Sackingen, Germany) to determine the patient’s teeth # 11 and 46 shades after calibrating it in accordance with the manufacturer’s instructions, as shown in Fig.1a. The students were then explained to use the VITA 3D-Master shade guide (Vita Zahnfabrik, Bad Sackingen, Germany) to choose the shade of the same teeth, 11 and 46, under clinic lightening condition and handheld corrected light (Tri-Shade, Zhengzhou, China) (Fig.1b &1c). The handheld corrected light was positioned 10 cm away from the shade tab that was being inspected. The ideal color temperature for choosing a shade is 5500°K, therefore in order to achieve precise shade matching, the lights was tuned to resemble daylight, which has a color temperature of about 5500K. The students were assigned to match each shade to the appropriate shade guide for each type of light source, one at a time. Four minutes was the time restriction per shade selection to reduce the possibility of errors due to extended times. The hue, chroma, and value of the color were recorded in predesigned form, following the manufacturer’s instructions (Fig.2).

### Ethical Approval:

The study was approved by the Research Ethics Committee at King Faisal University (KFU-REC-2023-OCT-ETHICS1318).

### Statistical Analysis:

The data was tabulated and subjected to statistical analysis using SPSS version 23.0. Quantitative variables were analyzed as means and standard deviations, whereas qualitative variables were reported as frequencies and percentages. The normality of the data was checked by the Shapiro-Wilks test. The Kruskal-Wallis test was applied to find the significant difference in shade selection between visual and instrumental methods with respect to academic years. A *p*-value of 0.05 or less is considered statistically significant.

## RESULTS

A total of 70 male undergraduate students participated in this study, with a mean age of 23.07±0.84 years. Among the study participants, 22(31.4%) were from the 4^th^ year, 25**(**35.7%) were from the 5^th^ year, and 23**(**32.9%) were from the 6^th^ year. All participants successfully passed the Ishihara test, suggesting a uniformity in color vision ability among the study group, as shown in Table-I.

**Table-I:** Demographic characteristics of participants (n =70).

Variable		Mean ± SD n(%)
Student’s Age (years)		23.07±0.84
Patient’s age (years)	15 - 30 years	59(84.3%)
	31 – 45 years	7(10.0%)
	46 - 60 years	4(5.7%)
Patient’s gender	Male	62(88.6%)
	Female	8(11.4%)
Students training level	4^th^ year	22(31.4%)
	5^th^ year	25(35.7%)
	6^th^ year	23(32.9%)
Ishihara test	Passed	70(100.0%)
	Failed	0(0.0%)

As far as the shade selection of tooth # 11 under correcting light is concerned, there was a statistically insignificant association observed among 4^th^, 5^th^, and 6^th^ year students with respect to the mean of the values 2.64±0.90, 2.36±0.95, and 2.39±0.72, respectively (p= 0.212). Moreover, a statistically insignificant association was noticed among 4^th^, 5^th^, and 6^th^ year students with respect to the mean of the hues of 2.09±0.61, 2.04±0.61, and 2.04±0.82, respectively (p= 0.777). Additionally, a statistically insignificant relationship was noticed among 4^th^, 5^th^, and 6^th^ year students with respect to the mean of the chroma of 2.18±1.18, 2.56±1.15, and 2.57±0.99, respectively (p= 0.434), as shown in Table-II.

**Table-II:** The association of shade selection of anterior tooth #11 by instrumental and visual methods under natural and correcting light with respect to the training level of students.

		Students training level			

Variables		4^th^ year	5^th^ year	6^th^ year	P-value (Kruskal-Wallis test)
Shade Selection by Spectrophotometer (Tooth # 11)	Value	3.09±1.19	3.12±1.16	3.00±1.08	0.948
	Hue	1.95±0.48	2.00±0.64	2.00±0.42	0.946
	Chroma	1.95±0.89	2.04±1.13	1.78±1.16	0.477
Shade Selection under Clinical lightening condition (Tooth # 11)	Value	2.55±0.85	2.20±0.86	2.57±0.78	0.089
	Hue	1.86±0.56	2.08±0.57	1.91±0.41	0.332
	Chroma	2.32±1.17	2.60±1.08	3.09±1.27	0.126
Shade Selection under correcting light (Tooth # 11)	Value	2.64±0.90	2.36±0.95	2.39±0.72	0.212
	Hue	2.09±0.61	2.04±0.61	2.04±0.82	0.777
	Chroma	2.18±1.18	2.56±1.15	2.57±0.99	0.434

The mean of the value of instrumental shade selection of tooth # 46 was slightly higher among 4^th^ year students (4.41±0.73), than that of 5^th^ year (3.72±1.02), and 6^th^ year students (3.83±0.83), with a statistically significant difference among them (p= 0.024), indicating 6^th^ year students selected darker shades possibly due to their higher training level. Additionally, a statistically significant relationship was noticed among 4^th^, 5^th^, and 6^th^ year students with respect to the means of the chroma 2.18 ±1.29, 2.92±1.11, and 3.13±1.10, respectively (p= 0.025), as shown in Table-III.

**Table-III:** The association of shade selection of posterior tooth # 46 by instrumental and visual methods under natural and correcting light with respect to the training level of students.

		Students training level			

Variables		4^th^ year	5^th^ year	6^th^ year	P-value (Kruskal-Wallis test)
Shade Selection by Spectrophotometer (Tooth # 46)	Value	4.41±0.73	3.72±1.02	3.83±0.83	0.024
	Hue	2.14±0.35	2.12±0.33	2.13±0.45	0.985
	Chroma	1.95±1.43	2.72±1.83	2.00±1.24	0.332
Shade Selection under Clinical lightening condition (Tooth # 46)	Value	2.64±0.65	2.44±0.96	2.65±0.64	0.171
	Hue	2.05±0.57	2.00±0.81	1.96±0.76	0.918
	Chroma	3.18±1.36	3.24±1.20	3.39±1.23	0.851
Shade Selection under correcting light (Tooth # 46)	Value	2.73±0.63	2.44±1.04	2.61±0.72	0.137
	Hue	2.23±0.68	2.36±0.63	2.04±0.70	0.284
	Chroma	2.18±1.29	2.92±1.11	3.13±1.10	0.025

## DISCUSSION

The present study was designed to evaluate the shade selection accuracy of male dental students under clinical lightening condition and correcting light and using a spectrophotometer with respect to color parameters. It was revealed that no significant differences were observed in the shade selection of the anterior tooth using clinical lightening, correcting light and a spectrophotometer by considering hue, value, and chroma among dental students (p > 0.05). As far as posterior tooth shade selection is concerned, a significant difference was noticed in terms of value using a spectrophotometer, indicating most of the 6^th^ year students selected darker shades as compared to lighter shades (p=0.024). Additionally, a significant association was also found with respect to the chroma, using correcting light for shade selection among dental students (p= 0.025).

These findings are partially consistent with the previous research, which assessed shade matching under clinical and handheld corrective lighting conditions and demonstrated a statistically significant difference between the two lighting systems. The results of this study showed that shade-matching scores under corrective light were substantially higher than those under dental operatory light. These results are consistent with the research by Rayyan^13^, which demonstrated that portable stable light source was an economically viable source of balanced light that increased the importance of a shade-matching setting. The results of the present study also corroborated those of Nakhei et al.^8^, who confirmed our findings that shade matching with a corrective light device produced better results than with natural or clinical light. Furthermore, these findings aligned with the investigations conducted by Corcodel et al.^7^ and Mete et al.^14^ Similarly, Siddique et al. also observed the superiority of digital shade selection method over the visual. The correct tooth shade was selected in 39.4% of cases by visual while 66% of cases by digital method.^15^

The level of education and training received in shade matching both show a strong correlation with shade matching performance.^16^-^17^ Previous research has shown that dental professionals need to participate in hands-on learning opportunities, continuing education initiatives, and additional instruction sessions in order to enhance their shade-matching skills.^4^,^18^ Another cross-sectional study examined the degree of precision of shade selection between clinical and non-clinical students and found a substantial difference (p < 0.05) between all shade tabs under correcting light between the two groups of students, demonstrating that clinical experience of color matching improves shade selection performance.^19^ As far as the present study is concerned, a significant difference was evident between all shade tabs with respect to value under spectrophotometer and with respect to chroma under correcting light among undergraduate’s dental students (p < 0.05) in the shade matching of the posterior tooth. Another study supported these findings, and their results urged dentists to aggressively pursue precise training for shade matching, incorporate color-corrected light equipment into their performances to choose the shade, and execute their abilities.^20^ Similarly, a further study corroborated the present study and found that students’ shade matching abilities under a color-correcting device improved shade selection in comparison to the traditional method under typical lighting circumstances.^21^

Color vision is an essential component of prosthodontics, restorative dentistry, and aesthetic dentistry because impaired color vision makes it harder to perceive color accurately than in healthy color vision dentists.^22^-^24^ As color vision impairment has been proven to cause inferior color matching quality, one study suggested that participants with any kind of color vision deficiency should be dismissed from studies.^25^ According to the present study, none of the students had any color vision imperfections, supporting these findings.

Considering the findings of our study, it is imperative to state that the shade selection in posterior teeth requires a thorough understanding of different color parameters and the application of these parameters in the clinical setting. By employing correct lightening conditions with a color temperature of between 5,500 K and 6,500 K and a Color Rendering Index (CRI) of at least 90 during the tooth shade selection process, the dentist can choose a tooth shade that is more accurate and closely matches the natural tooth color.

It is advised that more research be done to evaluate handheld corrected light (Tri-Shade, Zhengzhou, China) against alternative correcting lights and to carry out extensive randomized controlled clinical trials in order to gain a better understanding of various computerized techniques perform in order to achieve successful shade matching. Additionally, there are spectrophotometers on the market that need further investigation in order to provide a thorough grasp of shade matching between the restoration and the tooth.

### Limitations:

Some of the limitations of this study include the fact that shade tab matching was the only factor taken into account; more research is needed to match shade tabs to natural dentition and evaluate shade using other digital devices, like digital cameras, for shade measurement. It is advised to use a standard light source with a full spectrum and provide appropriate settings in the clinical context, within the constraints of this study. Nowadays, the majority of dental offices use artificial lighting, and many dental procedures are conducted in locations where daylight is frequently scarce. Because excessive lighting circumstances might negatively impact the shade matching procedure, it is imperative to set up a distinct area for this purpose. The study’s findings are also not generalizable because they only included male students with less clinical expertise.

## CONCLUSION

Academic years had a significant impact on the shade selection of posterior teeth by instrumental and visual methods with respect to color parameters. It is advisable that more attention should be paid on educational programs for tooth-shade matching, given the benefits of practical training and instruction. Dental students become more proficient at matching shades by incorporating clinical practice into dentistry programs.
